# Distinct neural markers of evidence accumulation index metacognitive processing before and after simple visual decisions

**DOI:** 10.1093/cercor/bhae179

**Published:** 2024-05-05

**Authors:** Caleb Stone, Jason B Mattingley, Stefan Bode, Dragan Rangelov

**Affiliations:** Queensland Brain Institute, QBI Building 79, The University of Queensland, St Lucia 4072, Queensland, Australia; Queensland Brain Institute, QBI Building 79, The University of Queensland, St Lucia 4072, Queensland, Australia; School of Psychology, McElwain Building 24A, The University of Queensland, St Lucia 4072, Queensland, Australia; Melbourne School of Psychological Sciences, Redmond Barry Building, The University of Melbourne, Parkville 3010, Victoria, Australia; Queensland Brain Institute, QBI Building 79, The University of Queensland, St Lucia 4072, Queensland, Australia; School of Economics, Colin Clark Building 39, The University of Queensland, St Lucia 4072, Queensland, Australia

**Keywords:** centro-parietal positivity, decision-making, electroencephalography, metacognition, motion discrimination

## Abstract

Perceptual decision-making is affected by uncertainty arising from the reliability of incoming sensory evidence (perceptual uncertainty) and the categorization of that evidence relative to a choice boundary (categorical uncertainty). Here, we investigated how these factors impact the temporal dynamics of evidence processing during decision-making and subsequent metacognitive judgments. Participants performed a motion discrimination task while electroencephalography was recorded. We manipulated perceptual uncertainty by varying motion coherence, and categorical uncertainty by varying the angular offset of motion signals relative to a criterion. After each trial, participants rated their desire to change their mind. High uncertainty impaired perceptual and metacognitive judgments and reduced the amplitude of the centro-parietal positivity, a neural marker of evidence accumulation. Coherence and offset affected the centro-parietal positivity at different time points, suggesting that perceptual and categorical uncertainty affect decision-making in sequential stages. Moreover, the centro-parietal positivity predicted participants’ metacognitive judgments: larger predecisional centro-parietal positivity amplitude was associated with less desire to change one’s mind, whereas larger postdecisional centro-parietal positivity amplitude was associated with greater desire to change one’s mind, but only following errors. These findings reveal a dissociation between predecisional and postdecisional evidence processing, suggesting that the CPP tracks potentially distinct cognitive processes before and after a decision.

## Introduction

Perceptual decision-making involves the detection, discrimination, and categorization of sensory information to guide behavior (Hanks and Summerfield 2017). These processes are affected by at least two sources of uncertainty (Bang and Fleming 2018). At the perceptual level, the reliability of incoming sensory information determines the precision of neural representations, with less reliable stimuli being associated with greater uncertainty (Yeung and Summerfield 2012; Bitzer and Kiebel 2015; Rangelov et al. 2021). Uncertainty can also arise from the categorization of internal representations relative to a category boundary. As the distance between the boundary and the encoded representation is reduced, uncertainty increases (Kepecs et al. 2008; Gold and Ding 2013; Hebart et al. 2016). Previous neuroimaging studies (Shankar and Kayser 2017;Bang and Fleming 2018; Bang et al. 2020) have used random dot motion paradigms to combine parametric manipulations of perceptual uncertainty (i.e. motion coherence) and categorical uncertainty (i.e. angular offset between motion direction and a criterion). In addition to reporting effects on behavioral performance (i.e. reaction time and accuracy), these studies found that perceptual and categorical uncertainty can be differentiated neurally (Bang and Fleming 2018; Bang et al. 2020) and are weighted differently during the decision process (Shankar and Kayser 2017). It remains unclear, however, if perceptual and categorical uncertainty also exert dissociable effects on the temporal dynamics of evidence processing during decision-making.

Along with reaction time and accuracy, previous research has explored the effects of perceptual and categorical uncertainty on decision-makers’ subjective evaluations of the quality of their choices. Such *metacognitive* behaviors (e.g. confidence ratings and changes of mind) reflect the combined readout of internal uncertainty computed from all sources, including the reliability of sensory evidence and its distance from the category boundary (Pouget et al. 2016; Bang and Fleming 2018; Boundy-Singer et al. 2022). This research has found that increased perceptual and/or categorical uncertainty leads to lower confidence ratings (Spence et al. 2015; Boldt et al. 2017; Bang and Fleming 2018; Bang et al. 2020) and more frequent changes of mind (Resulaj et al. 2009; Freeman 2014; Barca and Pezzulo 2015; Atiya et al. 2020) but has not considered the neural time course of these effects. By one account, metacognition is based on the same evidence used to inform the initial decision (Moran et al. 2015). Uncertainty may therefore impact metacognitive judgments via its effect on predecisional evidence processing. For example, the difference in accumulated evidence between choice alternatives (Vickers and Packer 1982) or the precision of the accumulated evidence (Yeung and Summerfield 2012) may be read out as a confidence rating. Alternatively, metacognition may be informed by postdecisional evidence processing, with uncertainty impacting metacognitive judgments via processing of new sensory information (Resulaj et al. 2009) or internally generated conflict signals (Desender et al. 2021). Disentangling these alternatives remains an ongoing topic of investigation as past research has reported inconsistent results (e.g. Feuerriegel et al. 2022; Grogan et al. 2023).

When investigating the temporal dynamics of evidence processing, past work has utilized the high temporal resolution of electroencephalography (EEG) to characterize neural correlates of evidence accumulation in the form of the centro-parietal positivity (CPP; O’Connell et al. 2012; Kelly and O’Connell 2013; Twomey et al. 2015; Rangelov and Mattingley 2020). The CPP varies with evidence reliability (Kelly and O’Connell 2013) and is associated with decision confidence (Herding et al. 2019; Rausch et al. 2020), but its sensitivity to distinct sources of uncertainty remains unclear. Here, we used the CPP to examine the effects of both perceptual and categorical uncertainty on the dynamics of evidence processing during decision-making and subsequent metacognitive judgments. We developed a motion discrimination task in which we varied motion coherence and angular offset of the motion direction from a criterion, allowing us to capture uncertainty related to the strength of internal stimulus representations (i.e. motion coherence) and the distance of those representations from the decision criterion (i.e. angular offset). After each response, we recorded participants’ desire to change their minds on a continuous scale, allowing us to quantify subjective uncertainty. Finally, we performed time-resolved analyses of the CPP in response to motion signals and response execution, allowing us to characterize the relationship between evidence processing and behavioral performance.

## Materials and methods

### Participants

Thirty-seven participants (26 females, M_age_ = 24.16 ± 5.17 years) were recruited via The University of Queensland’s research participation scheme in exchange for reimbursement (20 AUD/h). One participant was excluded due to an interrupted session, leaving a final sample of 36. Participants were aged 18 to 40 years and had self-reported normal or corrected-to-normal vision. Additional exclusion criteria included abnormal color vision, acute neurological illness, or drug use. The study was approved by The University of Queensland Human Research Ethics Committee (2021/HE000161) and participants provided written informed consent prior to participation.

### Apparatus

Participants were seated in a dark, acoustically, and electrically shielded room with their head stabilized by a chinrest at a viewing distance of 56 cm. Stimuli were displayed on a 22.5” VIEWPixx monitor with a resolution of 1,920 × 1,080 pixels and 100 Hz refresh rate. The task was coded using the PsychoPy toolbox in Python (Peirce 2007). EEG data were collected using a BioSemi ActiveTwo system from 64 Ag-AgCl electrodes placed according to the 10–20 system with a sampling frequency of 1024 Hz. Electrooculographic data were collected using electrodes placed above and below the left eye and on the outer canthi. Eye-movement data, which are not reported here, were collected using an EyeLink 1000 eye tracker (SR Research).

### Experimental design

**Fig. 1:**
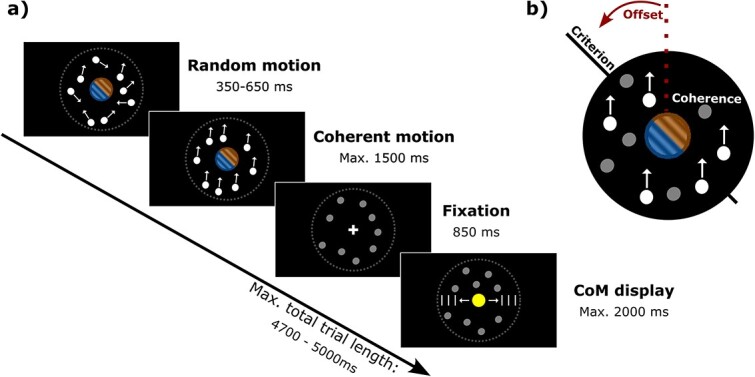
a) Example trial sequence from the motion discrimination task. Each trial began with a period of random dot motion randomly drawn from a uniform distribution of between 350 to 650 ms in 10 ms steps. Random motion was followed by coherent motion until response or for maximally 1500 ms. If a response was made, the dots stopped moving and remained stationary at a lower opacity for the remainder of the trial. A fixation cross was then displayed for 850 ms, after which the CoM display was shown until response or for maximally 2000 ms. b) The task manipulations. During the coherent motion period, the coherence of the dot motion (i.e. the probability that the dots would move in the specified direction) and its angular offset from the criterion orientation were pseudo-randomly selected per trial to induce greater uncertainty (i.e. low coherence and/or small offset) or lesser uncertainty (i.e. high coherence and/or large offset).

The task display consisted of 320 moving dots (diameter: 0.2 degrees of visual angle [dva]; dot life: 0.2 s; dot speed: 5 dva/s during random motion and 6.5 dva/s during coherent motion) within a circular display area (diameter: 15.6 dva). A black [RGB: 0, 0, 0] circular aperture was positioned at the center of the display area (diameter: 2.34 dva), and a colored, oriented Gabor patch (diameter: 1.2 dva; one-half blue [RGB: 0,127,255], the other orange [255, 128, 0]) was presented at the center of the aperture. No dots appeared within the aperture (see Fig. 1). On each trial, participants were required to report, as quickly and as accurately as possible, whether the dots moved toward the orange or the blue side of the Gabor using the left and right mouse buttons (color-key mapping was counterbalanced between participants). Participants were instructed to maintain fixation on the Gabor patch throughout the trial.

Each trial began with random dot motion for 0.35 to 0.65 s to prevent evoked responses from stimulus onset contaminating EEG epochs of interest. A period of coherent motion lasting for maximally 1.5 s was then presented, during which time participants could register a response. If participants did not respond, a “!” appeared in place of the Gabor for 0.75 s. Coherent dot-motion signals were generated by randomly and independently sampling the displacement of each dot between 2 frames from a von Mises distribution defined by 2 experimentally manipulated parameters. The location parameter (μ) defined the central value of the distribution, or the average motion direction, whereas the concentration parameter (K) defined the width of the distribution, or the motion coherence. On each trial, the average motion direction (μ) was pseudo-randomly offset by either ±30° (large) or ±15° (small) relative to the Gabor’s orientation (i.e. the criterion). The criterion orientation was pseudo-randomly selected from 1 of 4 values (i.e. 0/180°, 45/225°, 90/270°, or 135/315°) and remained constant within a block of trials. Thus, if the criterion orientation was 0/180°, the possible motion directions in that block were −30°, −15°, 15°, 30°, 150°, 165°, 195°, or 210°, which were counter-balanced within that block. The coherence value was pseudo-randomly selected per trial, varying between high (K = 0.66) and low (K = 0.44). The experimental design was thus a 2 (motion coherence: low and high) × 2 (offset value: small and large) fully within-subjects design. Criterion orientation was varied as a control variable but was not of primary interest for analyses.

Following each response, a fixation cross was displayed for 0.85 s, followed by a change-of-mind (CoM) display presented for 2 s (maximum). The CoM display consisted of a Likert scale with 10 white, vertical notches placed on either side of a question mark (“?”) centered in the middle of the display space. Participants were instructed to report *to what extent* they would like to change their mind regarding their response about motion direction. To do so, participants positioned a yellow response disk via the computer mouse along the horizontal axis of the display, with the right-most position of the scale anchored by “completely” and the left-most position anchored by “not at all”. Responses were recorded by pressing the left mouse button. The use of this scale allowed us to capture a bidirectional readout of participants’ subjective uncertainty in their decision. The next trial began immediately after the response or if participants failed to respond within the 2 s limit. Participants completed 8 blocks of 20 practice trials (160 in total), followed by 16 blocks of 64 experimental trials (1,024 in total).

## Statistical analyses

### Behavioral analyses

Trials with missing motion direction responses (5.2% of all trials) or responses faster than 0.2 s (less than 1% of responses) were removed from further analysis. Change of mind scores were first Z-scored per participant to remove the influence of individual response bias for or against changes. Trials with Z-scores greater than 5 or less than −5 were excluded (1.4% of trials). To assess metacognitive sensitivity, we subtracted Z-scored change of mind values on correct trials from those on error trials. Larger difference scores reflect greater separation between correct and error responses, or better metacognitive sensitivity. Reaction times for correct responses and transformed CoM scores were analyzed using general linear mixed-effects models with maximum likelihood estimation using the lme4 package (Bates et al. 2015) in the R statistical programming language. Accuracy data were also modeled with the lme4 package, but using a generalized linear mixed-effects model with a logit link function and a binomial conditional distribution (Jaeger 2008; Brown 2021). All models were run on single-trial data except for those involving CoM difference scores. For each dependent variable (DV), the model included coherence, offset, and their interaction as fixed-effect predictors and per-participant random intercepts:


$$ \mathrm{DV}\sim 1+\mathrm{Coherence}\ast \mathrm{Offset}+\left(1|\mathrm{Participant}\_\mathrm{ID}\right). $$

For completeness, we ran an additional model for each DV including the effect of criterion orientation (i.e. cardinal vs. oblique) and all higher-order interactions. Inclusion of criterion orientation did not alter the conclusions for any DV, and so is not reported in text.

### E‌EG analyses

EEG data were preprocessed using the MNE toolbox in Python (Gramfort et al. 2013). Data were re-referenced offline to the average of all electrodes, band-pass filtered between 0.1 to 99 Hz and notch filtered at 50 Hz to remove line noise. The data then underwent automated artifact rejection using the FASTER algorithm (Nolan et al. 2010). The continuous data were then segmented into epochs time-locked to the onset of the coherent motion signal (−0.1 to 0.9 s). These epochs were baseline-corrected relative the −0.1 to 0.0 s period prestimulus and linearly detrended. Response-locked epochs were created by aligning the stimulus-locked data to the response onset (−0.3 to 0.7 s), enabling us to use the same prestimulus baseline correction. The segmented data were down-sampled to 256 Hz and bad epochs were identified and removed using the FASTER algorithm (*M/SD* = 47/17 across participants; range: 11 to 95).

For analysis of the CPP, we selected a cluster of 5 centro-parietal electrodes centered on CPz (i.e. Cz, CPz, Pz, CP1, and CP2) and averaged across these electrodes to increase signal-to-noise ratio. This cluster was chosen to align with the location previously reported in studies of the CPP (e.g. O’Connell et al. 2012; Kelly and O’Connell 2013; Rangelov and Mattingley 2020). Of note, other event-related potential (ERP) components have been reported in the literature as an index of evidence accumulation, including the predecisional P3b (Twomey et al. 2015; Rausch et al. 2020) and the postdecisional error positivity (Pe; Steinhauser and Yeung 2012; Boldt and Yeung 2015). Despite significant differences between these components (e.g. being either stimulus- or response-locked), they overlap considerably, share polarity, and yield similar topographies/latencies (Ullsperger et al. 2010; O’Connell et al. 2012). For simplicity, we thus refer to ERPs derived from the centro-parietal electrode cluster as the predecisional and postdecisional CPP. In addition to the CPP analyses, we also analyzed ERPs from a second, fronto-central cluster of electrodes centered on FCz (i.e. Fz, FCz, Cz, FC1, and FC2). This cluster was based on inspection of stimulus-locked topographies, which revealed a fronto-central positivity at this region. Both electrode clusters were analyzed per time sample using single-trial general linear mixed-effects models with fixed effects for coherence, offset, and their interaction, and by-participant random intercepts:


$$\mathrm{Amplitude}\sim 1+ \mathrm{Offset}\ast \mathrm{Coherence}+\left(1|\mathrm{ Participant\_ ID}\right)$$

False discovery rate (FDR) corrections across time samples were applied to control for multiple comparisons (Benjamini and Hochberg 1995).

To investigate whether, and at which time points, the CPP predicted metacognitive judgments, we modeled CoM scores as a function of trial-wise CPP amplitude. Since manipulation of perceptual and categorical uncertainty were expected to impact both CPP amplitude and CoM scores, potential correlations between the two could have originated, trivially, from the experimental manipulations. To independently examine the correlation, we first removed the variability generated by experimental manipulations by regressing CPP amplitude per time sample onto coherence and offset in a trial-wise general linear model and extracted the residuals from these models:


$$ \mathrm{Amplitude}\sim 1+\mathrm{Offset}\ast \mathrm{Coherence} $$

This analysis was conducted per participant to account for individual differences in the effects of coherence and offset. The residual CPP amplitude reflects trial-wise variability unrelated to coherence and offset manipulations, albeit still influenced by variations in sensory input due to random fluctuations of the stimuli. The residuals were then Z-scored per participant and entered as predictors in separate time-resolved, trial-wise general linear mixed-effects models predicting CoM scores from CPP residuals. Moreover, to determine if the relationship between CPP residuals and CoM scores depended on initial response accuracy, we included a fixed effect of response accuracy and its interaction with CPP residuals. The final model formula was as follows:


$$ \mathrm{CoM}\ \mathrm{score}\sim 1+\mathrm{CPP}\_\mathrm{residuals}\ast \mathrm{accuracy}+\left(1|\mathrm{Participant}\_\mathrm{ID}\right) $$

## Results

### Behavior

**Fig. 2:**
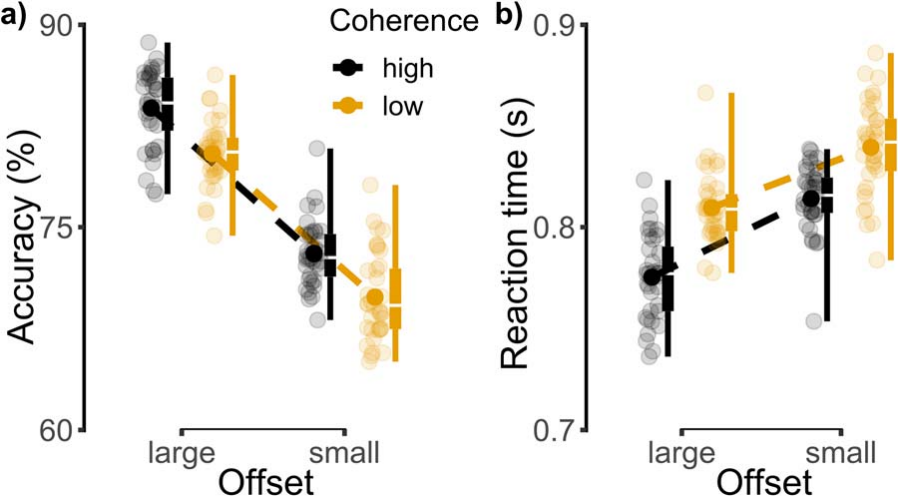
Boxplots of a) accuracy and b) reaction time split by coherence and offset conditions. In both panels, filled circles denote the mean and semi-transparent circles denote individual participants. For visualization, between-participant variability was removed using previously described methods (Morey 2008).

We first examined the effects of coherence and offset on participants’ motion discrimination decisions (see Fig. 2). As expected, response accuracy was lower as uncertainty increased, with both the effects of coherence (*B* = −0.244; *SE* = 0.041; *z*(34787) = −6.0; *P* < 0.001) and offset (*B* = −0.689; *SE* = 0.039; *z*(34787) = −17.73; *P* < 0.001) being significant. That is, trials with a smaller offset and low coherence significantly decreased accuracy relative to large offset, high coherence trials. The interaction between coherence and offset was not significant (*B* = 0.074; *SE* = 0.054; *z*(34787) = 1.39; *P* = 0.166). Reaction times became longer as uncertainty increased, with the effects of coherence (*B* = 0.035; *SE* = 0.003; *t*(26718) = 10.52; *P* < 0.001) and offset (*B* = 0.042; *SE* = 0.003; *t*(26718) = 12.25; *P* < 0.001) again being significant. The interaction between coherence and offset was only marginally significant (*B* = **−**0.009; *SE* = 0.005; *t*(26718) = −1.86; *P* = 0.064), with a slightly stronger effect of offset for high coherence relative to low coherence trials.

**Fig. 3:**
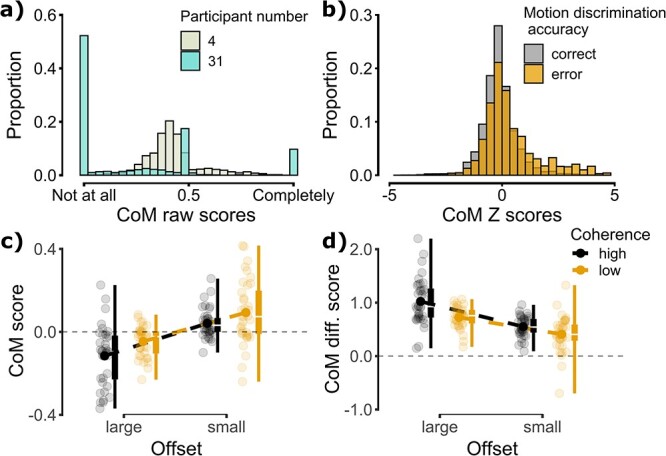
a) Histograms of raw CoM scores for 2 example participants, highlighting substantial individual differences. b) Histogram of Z-scored CoM scores for error (orange) and correct (gray) trials. c) Boxplots of Z-scored CoM scores split by coherence and offset conditions. d) Boxplots of Z-scored CoM differences (error − correct) split by coherence and offset conditions. Note that in panels c and d conventions are as in Fig. 2.

We next examined the effects of perceptual and categorical uncertainty on participants’ willingness to change their mind. Given the use of a continuous CoM measure, we first looked at the distribution of CoM scores. As shown in Fig. 3a, there was significant heterogeneity in participants’ use of the CoM scale, with some participants expressing a much greater desire to change their motion discrimination responses than others. To remove these individual biases, the raw CoM responses were Z-scored per participant, such that positive values indicate a stronger desire to change one’s mind relative to the participant-specific average (Fig. 3b). Z-scored CoM scores are reported for all further analyses. Overall, greater uncertainty increased participants’ desire to change their mind, with CoM scores being positive for low coherence trials and negative for high coherence trials (*M/SD =* 0.023/0.077 vs. **−**0.038/0.074) and positive for small offset trials and negative for large offset trials (*M/SD* = 0.067/0.104 vs. **−**0.081/0.097; see Fig. 3c). In line with this, the effects of coherence *(B* = 0.070; *SE* = 0.014; *t*(34285) = 4.94; *P* < 0.001) and offset (*B* = 0.157; *SE* = 0.014; *t*(34284) = 11.12; *P* < 0.001) were both significant. The interaction between coherence and offset was not significant (*B* = −0.016; *SE* = 0.020; *t*(34282) = −0.80; *P* = 0.424).

While these results reveal that greater uncertainty increased participants’ desire to change their mind overall, it is important to consider the relationship between CoM scores and the accuracy of participants’ motion discriminations. We first confirmed that CoM scores were higher following errors than correct responses (*M/SD =* 0.500/0.556 vs. **−**0.118/0.112; *B* = 0.527; *SE* = 0.012; *t*(30076) = 44.09; *P* < 0.001). Next, we computed difference scores by subtracting CoM scores following correct responses from those following error responses. Larger difference scores indicate greater metacognitive sensitivity. We again found that the effects of coherence (*B* = −0.376; *SE* = 0.092; *t*(252) = −4.09; *P* < 0.001) and offset (*B* = −0.539; *SE* = 0.092; *t*(252) = −5.86; *P* < 0.001; Fig. 3d) were significant, such that the separation between correct and error responses was greater when uncertainty was low. Thus, although uncertainty increased participants’ desire to change their mind, it reduced their ability to do so accurately. Finally, the interaction between coherence and offset was not significant (*B* = 0.198; *SE* = 0.130; *t*(252) = 1.52; *P* = 0.129).

### E‌EG

**Fig. 4:**
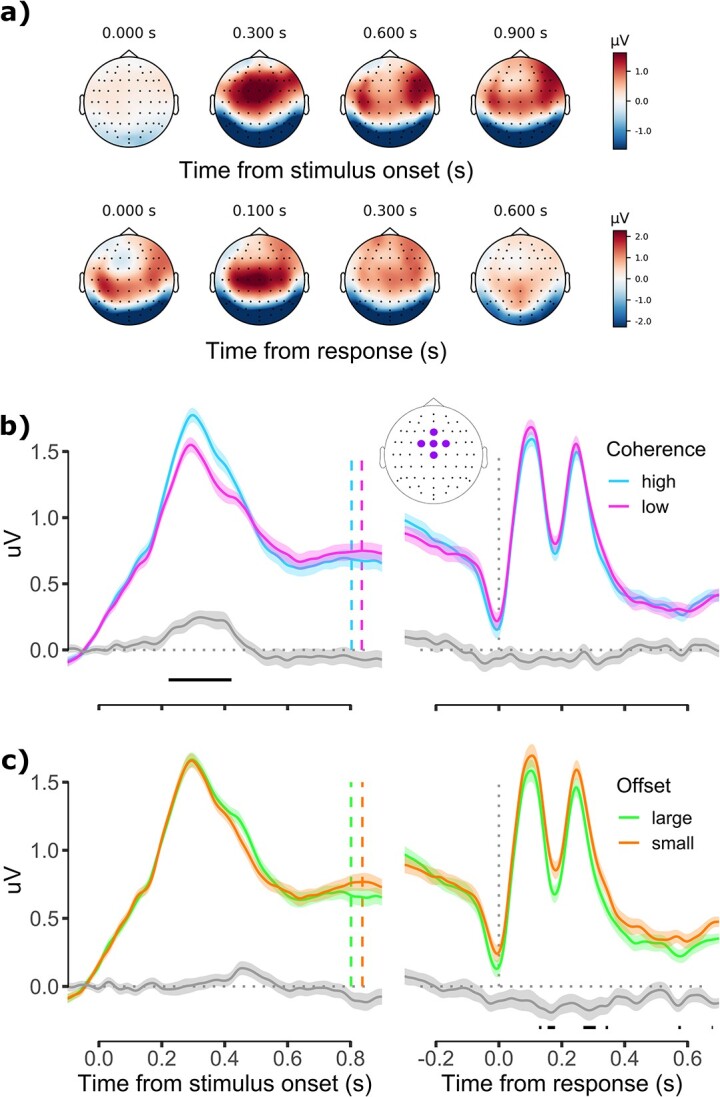
a) Topographic maps of EEG activity during the stimulus-locked (top) and response-locked (bottom) epochs. b) Fronto-central positivity spilt by coherence for stimulus-locked (left) and response-locked (right) epochs. Inset shows electrode cluster used for analysis (i.e. Fz, FCz, Cz, FC1, and FC2). c) Same as b but split by offset. In all panels, the gray line shows the difference between conditions with standard errors. Vertical colored lines in stimulus-locked epochs denote mean reaction time for the color-matched condition in legend. Vertical dashed lines in response-locked epochs denote response time. Horizontal black lines denote periods of statistical significance for fixed effects of condition (*P* < 0.05; residual degrees of freedom for stimulus- and response-locked models were 35,122 and 33,149, respectively), FDR corrected for multiple comparisons. Error bands denote normalized 95% confidence intervals as per (Morey 2008). Data were smoothed using a Gaussian window (SD = 16 ms) for visualization only.

Having established that greater uncertainty impaired both the quality of participants’ initial decisions and their ability to reflect on those decisions, we next explored how uncertainty affected neural markers relevant to decision formation. Inspection of stimulus-locked topographies (see Fig. 4a) revealed a broad fronto-central positivity centered on FCz which peaked at about 300 ms post-stimulus onset. This topography is broadly consistent with the P3a, a positive deflection occurring over frontal electrodes which is thought to reflect stimulus-driven attentional mechanisms (Polich 2007; Yau et al. 2021; Botelho et al. 2023). Consistent with the hypothesized functional role of P3a, analysis of the fronto-central component revealed an amplitude modulation by coherence from about 220 to 420 ms post-stimulus, with higher coherence associated with a larger amplitude (see Fig. 4b, left panel). There were no significant main effects of offset at any time point during the stimulus-locked epochs (Fig. 4c, left panel). In response-locked epochs, the amplitude of fronto-central component decreased prior to response, which is consistent with previous studies (Kelly and O’Connell 2013; Feuerriegel et al. 2022) and possibly reflects motor preparation and execution (Schurger et al. 2021). Analyses revealed no significant main effect of coherence during this time period (Fig. 4b, right panel), but a significant main effect of offset at several time points, with overall higher positivity for small offset trials than large offset trials (see Fig. 4c, right panel). There were no periods in either the stimulus- or response-locked epochs during which the interaction between coherence and offset was significant (see Supplementary Fig. S1). Overall, these analyses showed the onset of the fronto-central component to coincide with the stimulus onset and its amplitude to decrease prior to response, suggesting that this component most likely does not reflect the temporal dynamics of evidence accumulation, which is expected to have a delayed onset and to increase in amplitude prior to response (O’Connell et al. 2012; Kelly and O’Connell 2013).

**Fig. 5:**
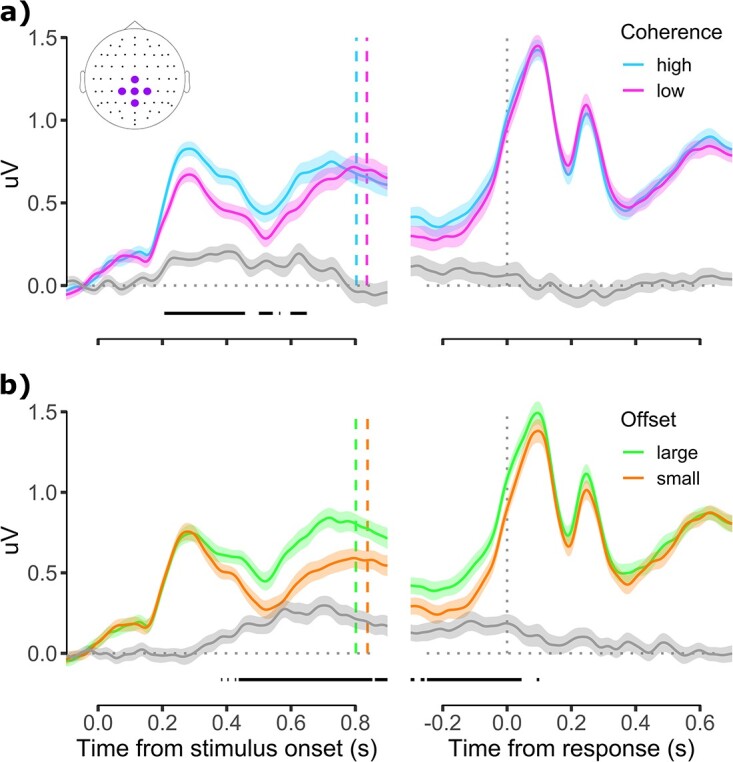
a) CPP spilt by coherence for stimulus-locked (left) and response-locked (right) epochs. Inset shows electrode cluster used for analysis (i.e. Cz, CPz, Pz, CP1, and CP2). b) Same as a but split by offset. Conventions as in Fig. 4.

Next, we characterized the time-course of a more posterior, centro-parietal cluster of electrodes. When examining ERPs at this cluster (see Materials and methods), we observed a positive deflection beginning about 180 ms after stimulus onset, followed by a second positive deflection beginning about 500 ms after stimulus onset whose peak latency aligned approximately with mean response times, consistent with the CPP (see Fig. 5). These 2 deflections were differentially sensitive to the coherence and offset manipulations. As shown in Fig. 5a, when split by coherence, the first deflection reached a significantly greater amplitude during high relative to low coherence trials, and this was carried over for periods of the second deflection. In contrast, when split by offset, large offset trials reached a significantly greater amplitude than small offset trials during the decline of the first deflection and throughout the second deflection (see Fig. 5b, left panel). When considering the response-locked epochs, we observed build-to-threshold dynamics consistent with previous literature (O’Connell et al. 2012; Kelly and O’Connell 2013), with amplitude being significantly greater for large relative to small offset trials until about 50 ms post-response. However, there were no amplitude differences between high and low coherence trials. Moreover, we observed a second, smaller positive deflection peaking at about 300 ms post-response, and a third, final positive deflection rising from about 400 to 600 ms post-response. The second deflection at about 300 ms may reflect a visual evoked potential resulting from the change in stimulus display as following the response the dots stopped moving and the fixation cross appeared on screen. The third deflection may reflect the start of a second, postdecisional evidence accumulation process as has been proposed previously (Steinhauser and Yeung 2012; Murphy et al. 2015; Desender et al. 2021). In the absence of new stimulus information, this evidence may have arisen from, for example, residual evidence in the sensory processing pipeline or internal cues such as memory traces or conflict signals (Resulaj et al. 2009; Desender et al. 2021). There were no periods in either the stimulus- or response-locked epochs during which the interaction between coherence and offset was significant (see Supplementary Fig. S2).

### Relationship between the CPP and CoM responses

**Fig. 6:**
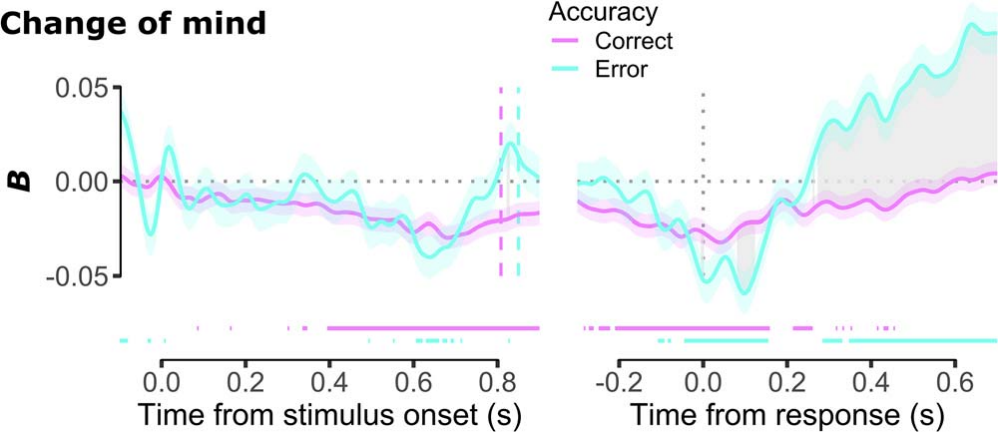
Trial-wise regressions of CoM score onto residual CPP amplitude and response accuracy after having regressed out the influence of coherence and offset for stimulus-locked (left) and response-locked (right) epochs. Error bars denote standard error. Horizontal colored bars denote periods of statistical significance for fixed-effect of residual CPP amplitude at the color-matched level of response accuracy (*P* < 0.05; residual degrees of freedom for stimulus- and response-locked models were 32,835 and 32,757, respectively). Gray shading denotes periods of significant interaction between response accuracy and residual CPP amplitude. All statistical tests are FDR corrected for multiple comparisons.

In a time-resolved, trial-wise analysis, we next regressed CoM scores onto response accuracy and residual CPP amplitude after having regressed out the influence of coherence and offset (see Materials and methods). Starting from about 400 ms after stimulus onset (Fig. 6, left panel), and coinciding with the onset on the second CPP deflection (see Fig. 5), the CPP was a significant negative predictor of CoM scores on correct trials such that higher CPP amplitude was associated with less desire to change one’s mind. On incorrect trials, the relationship between the CPP and CoM scores was much less consistent in time and polarity. It was negative from about 600 to 700 ms post stimulus onset, and for several other time periods throughout the epoch. There were, however, some time periods in which the relationship was positive, occurring during the baseline period and immediately prior to the response. When considering response-locked epochs, the negative relationship between the CPP and CoM scores for correct trials was present consistently until about 180 ms post-response, and for several other periods thereafter. On error trials, by contrast, the CPP predicted lower CoM scores for a period of about 200 ms around the time of the response, but this relationship reversed from about 300 ms post-response until the end of the epoch, such that larger CPP amplitude was associated with a *greater* desire to change one’s mind. This time window coincides with the peak of the third positive deflection observed in the response-locked CPP analysis (see Fig. 5). Moreover, there were multiple periods in which the interaction between CPP amplitude and response accuracy was significant, with the CPP on error trials being a significantly more negative predictor of CoM scores than on correct trials from about 100 to 150 ms post-response, and a significantly more positive predictor from about 300 ms post-response. Overall, these analyses revealed that the CPP amplitude is a significant predictor of subsequent changes of mind and that the response accuracy modulates the polarity (positive vs. negative) of this relationship.

## Discussion

Decisions are often made in the face of uncertainty. Here, we utilized the high temporal resolution of EEG recording to investigate how distinct sources of uncertainty related to the reliability of sensory evidence (i.e. perceptual uncertainty) and distance of that evidence from a category boundary (i.e. categorical uncertainty) impacted the temporal dynamics of evidence processing during perceptual decision-making. Moreover, we examined the time course of the relationship between evidence processing and participants’ metacognitive judgments.

### Predecisional CPP dissociates perceptual and categorical uncertainty

To manipulate perceptual and categorical uncertainty, we used a motion discrimination paradigm in which we varied motion coherence and angular offset of dot motion from a choice boundary, respectively. In line with previous studies (Shankar and Kayser 2017; Bang and Fleming 2018; Bang et al. 2020), we found that low coherence and small offsets impaired participants’ motion discrimination performance, leading to slower and less accurate choices. Poorer behavioral performance for low coherence and small offset trials was accompanied by modulations in decision-related ERP components. Specifically, we observed a stimulus-locked fronto-central component with a topography and latency consistent with the P3a (Polich 2007; Yau et al. 2021; Botelho et al. 2023) which was larger on high coherence trials. As this component was modulated by motion coherence and not angular offset, it may reflect enhanced attentional capture caused by the transition from random motion to high-coherence motion. While this component may reflect decision-relevant stimulus processing, the fact that it decreased and plateaued for the final about 300 ms of the stimulus-locked epoch and decreased in amplitude in the response-locked epoch makes it inconsistent with a neural decision variable (O’Connell et al. 2012; Kelly and O’Connell 2013; Twomey et al. 2015). As such, we analyzed ERPs from a cluster of centro-parietal electrodes and identified a dual-peaked CPP waveform. Like the fronto-central component, the CPP was larger in amplitude on high coherence trials, but also differentiated between large and small offset trials.

In contrast to previous studies (O’Connell et al. 2012; Kelly and O’Connell 2013; Rangelov and Mattingley 2020; Rangelov et al. 2021; Grogan et al. 2023), the predecisional, stimulus-locked CPP waveform was characterized by 2 distinct deflections, and different types of uncertainty affected these 2 deflections in distinct ways. Specifically, the first deflection occurred about 180 ms post-stimulus onset and was strongly modulated by motion coherence. By contrast, the second deflection was primarily modulated by offset and was closely aligned with the average reaction time. Given that our task design combined coherence and offset manipulations, the observed CPP waveform may reflect sequential processing of distinct aspects of uncertainty within a single decision interval. Specifically, the first deflection might reflect evidence accumulation for the *absolute* motion direction (i.e. the direction of the coherently moving dots), whereas the second deflection might reflect evidence accumulation regarding the *relative* motion direction (i.e. whether the coherent motion signal was to 1 side or the other of the criterion). Alternatively, given the proximity between the fronto-central and centro-parietal electrode clusters we analyzed, the first deflection may instead reflect the attentional processing mechanisms of the P3a influencing more posterior electrode sites via volume conduction (Feuerriegel et al. 2022). In this case, it may be that only the second CPP deflection reflects evidence accumulation per se. Supporting this alternative explanation, a defining characteristic of a neural decision variable is its systematic influence on behavior (Kelly and O’Connell 2015), and the period in which CPP amplitude reliably predicted CoM scores in our regression analysis coincided with the second stimulus-locked deflection only. This finding suggests that the first deflection may not reflect the decision variable as it did not exert a reliable influence on behavior.

### Differential influence of pre- and postdecisional brain activity on metacognition

Previous studies have proposed that the brain uses estimates of both perceptual and categorical uncertainty to inform metacognitive judgments (Yeung and Summerfield 2012; Bitzer and Kiebel 2015; Boldt et al. 2017; Bang and Fleming 2018; Boundy-Singer et al. 2022). In line with this past research, here, we observed that participants’ desire to change their mind increased additively with low coherence and small offsets. However, while uncertainty increased participants’ desire to change their mind, the separation between CoM scores following correct and error responses decreased with uncertainty, reflecting poorer metacognitive sensitivity with worse motion discrimination performance (Fleming and Lau 2014).

Past research has debated the role of pre- versus postdecisional evidence in contributing to metacognitive judgments (Steinhauser and Yeung 2010; Moran et al. 2015). Some theories propose that metacognition is informed by the same information that contributes to the decision process itself (Vickers and Packer 1982; Kiani et al. 2014; Van Den Berg et al. 2016), while others propose that evidence processing continues after a response is made, and it is the latter evidence that principally determines metacognitive judgments (Resulaj et al. 2009; Pleskac and Busemeyer 2010). Contrary to these proposals, we found that CPP amplitude exhibited a further postdecisional build-up after the response time, and that *both* predecisional and postdecisional CPP components predicted metacognitive judgments. Importantly, however, the strength and direction of this relationship depended on response accuracy. CPP amplitude on correct trials was exclusively negatively associated with CoM scores, and this relationship was principally reliant on predecisional evidence. By contrast, the negative association between predecisional CPP and CoM scores was much less consistent on error trials, but greater postdecisional CPP amplitude was associated with *higher* CoM scores on error trials. Our results therefore converge with studies that have highlighted the importance of considering both predecisional and postdecisional evidence processing (Murphy et al. 2015; Feuerriegel et al. 2022; Turner et al. 2022; Grogan et al. 2023). Importantly, however, our results go beyond past research by demonstrating an explicit sign reversal of the relationship between the CPP and metacognitive judgments as a decision unfolds over time.

The sign reversal in the relationship between CPP and metacognitive judgments raises important theoretical questions about the content of the evidence accumulated postdecisionally, and whether this differs from evidence accumulated predecisionally. Prior to a response, evidence accumulation is thought to map sensory information to choice alternatives (Ratcliff et al. 2016; Desender et al. 2021). Stronger sensory evidence therefore leads to greater evidence accumulation and higher confidence in the correctness of one’s choice (Herding et al. 2019; Rausch et al. 2020; Grogan et al. 2023). Once a response is made, however, it has been proposed that newly arriving evidence samples are instead interpreted as being either consistent or inconsistent with the original choice, and inconsistent evidence (i.e. evidence for having made a mistake) is selectively accumulated (Fleming et al. 2018; Desender et al. 2021). For example, in one study, a postdecisional CPP was found only on trials identified as errors (Murphy et al. 2015), suggesting that when participants were correct, or *thought* that they were correct, there was no inconsistent evidence to be accumulated. Similarly, other studies have found that greater Pe amplitude is associated with lower confidence (Boldt and Yeung 2015; Feuerriegel et al. 2022), again suggesting that postdecisional evidence accumulation leads participants to feel that they have erred.

Our results provide mixed support for the above proposal. On the one hand, we did not observe an amplitude difference between coherence or offset conditions during the third response-locked deflection. Given that participants committed more errors and had higher CoM scores on low coherence and small offset trials, we might have expected to see greater amplitude on these trials if postdecisional evidence accumulation is indeed selective for evidence of having made a mistake. On the other hand, although we saw no difference in the CPP waveforms postdecisionally, we observed that during the third response-locked deflection, there was a positive relationship between the CPP and CoM scores exclusively on error trials. By contrast, on correct trials, the negative relationship between the CPP and CoM scores returned to baseline by about 200 ms post-response. This pattern of results suggests that, even if the postdecisional evidence accumulation stage is not selective for inconsistent evidence (i.e. as suggested by the lack of any difference the CPP waveforms), the accumulated evidence may be selectively utilized to inform metacognitive judgments (i.e. as suggested by the difference in relationship between the CPP and CoM scores on correct and error trials).

Another intriguing theoretical question concerns the source of the postdecisional evidence that is accumulated in support of metacognitive judgments. In the current study, motion signals were extinguished at the time of response, suggesting that, beyond any residual evidence from the sensory buffers (Resulaj et al. 2009), alternative evidence sources likely contributed to the postdecisional CPP we observed. One plausible alternative evidence source is internally generated conflict signals (Murphy et al. 2015; Stone et al. 2022). Areas of the prefrontal cortex are purported to monitor performance and signal the need for cognitive control in the presence of conflict arising from competing stimulus or response representations (Botvinick et al. 2001; Cavanagh et al. 2009; Wendelken et al. 2009). In one study, stronger fronto-central theta power, a neural correlate of prefrontal activity, was found to predict error reports (Murphy et al. 2015). The authors thus concluded that metacognitive judgments are not only based exclusively on sensory evidence but also rely on high-order signals. Interestingly, as such signals do not strictly reflect information about the choice alternatives (e.g. neural activity encoding leftward or rightward motion), and are proportional to the amount of response conflict, they may be more easily interpreted as “error evidence” (Desender et al. 2021), as discussed above. Future work may explore the contribution of such prefrontal activity to the postdecisional CPP on a random dot motion paradigm such as ours, as was done by Murphy et al. (2015) using an error detection task.

## Conclusion

Here we have shown that distinct forms of uncertainty, reflecting perceptual processing and higher-order categorization, combine to impair decision-making and reduce metacognitive insight into decision quality. Moreover, we found that neural correlates of perceptual and categorical uncertainty have distinct time courses, possibly reflecting sequential processing stages within a single decision interval. Finally, the CPP was predictive of subsequent metacognitive judgments. Critically, this relationship was negative during predecisional period and positive during the postdecisional period. This finding identifies the CPP as a common neural signature of evidence accumulation for the initial choice as well as for metacognitive judgments, capable of tracking potentially distinct sources of evidence both before and after a perceptual decision is made.

## Authors contributions

Caleb Stone (Conceptualization, Data curation, Formal analysis, Investigation, Project administration, Visualization, Writing—original draft, Writing—review & editing), Jason B. Mattingley (Conceptualization, Funding acquisition, Methodology, Project administration, Resources, Supervision, Writing—review & editing), Stefan Bode (Conceptualization, Funding acquisition, Methodology, Writing—review & editing), and Dragan Rangelov (Conceptualization, Funding acquisition, Methodology, Project administration, Resources, Software, Supervision, Writing—review & editing).

## Funding

This work was supported by a grant from the Australian Research Network for Undersea Decision Superiority to D.R., J.B.M, and S.B (MYIP 10013).

##  

*Conflict of interest statement:* None declared.

## Data availability

The data underlying this article are available in https://osf.io/5ba2g/?view_only=f8091f101b4b4254aa0defef049704f3.

## Supplementary Material

Stone_et_al_2024_CerebralCortex_SupplementaryMaterial_final_bhae179
