# Supplementary material for: Distinct neural markers of evidence accumulation index metacognitive processing before and after simple visual decisions
Source: Cereb Cortex. 2024 May 5;34(5):bhae179. doi: 10.1093/cercor/bhae179 (PMC11070453; doi:10.1093/cercor/bhae179)
Supplement: Stone_et_al_2024_CerebralCortex_SupplementaryMaterial_final_bhae179 [file stone_et_al_2024_cerebralcortex_supplementarymaterial_final_bhae179.docx]

**Distinct neural markers of evidence accumulation index metacognitive processing before and after simple visual decisions**

**Supplementary Material**

Caleb Stone^1^, Jason B. Mattingley^1,2^, Stefan Bode^3^ Dragan Rangelov^1,4^

^1^Queensland Brain Institute, The University of Queensland, St Lucia 4072, Queensland, Australia

^2^School of Psychology, The University of Queensland, St Lucia 4072, Queensland, Australia

^3^Melbourne School of Psychological Sciences, The University of Melbourne, Parkville 3010, Victoria, Australia

^4^School of Economics, The University of Queensland, St Lucia 4072, Queensland, Australia

Corresponding Author: Dragan Rangelov, +61 7 334 67059, [d.rangelov@uq.edu.au](mailto:d.rangelov@uq.edu.au), QBI Building 79, University of Queensland, St Lucia QLD 4067


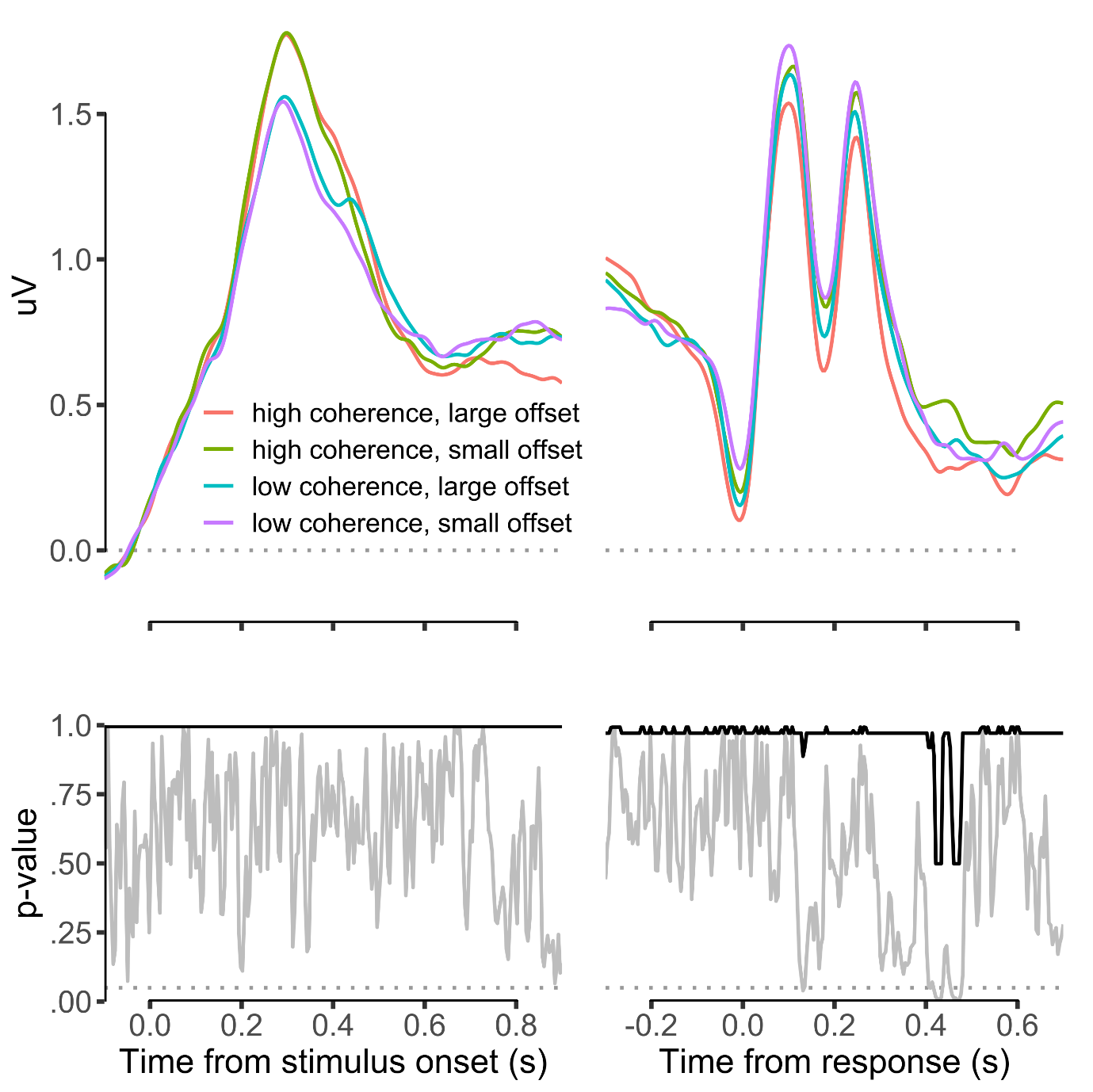


***Figure S1.*** Fronto-central P3a (Fz, FCz, Cz, FC1, and FC2 electrodes) spilt by Coherence and Offset for stimulus-locked (left) and response-locked (right) epochs. Lower panels show raw (grey) and false-discovery rate corrected (black) p-values for the interaction effect.


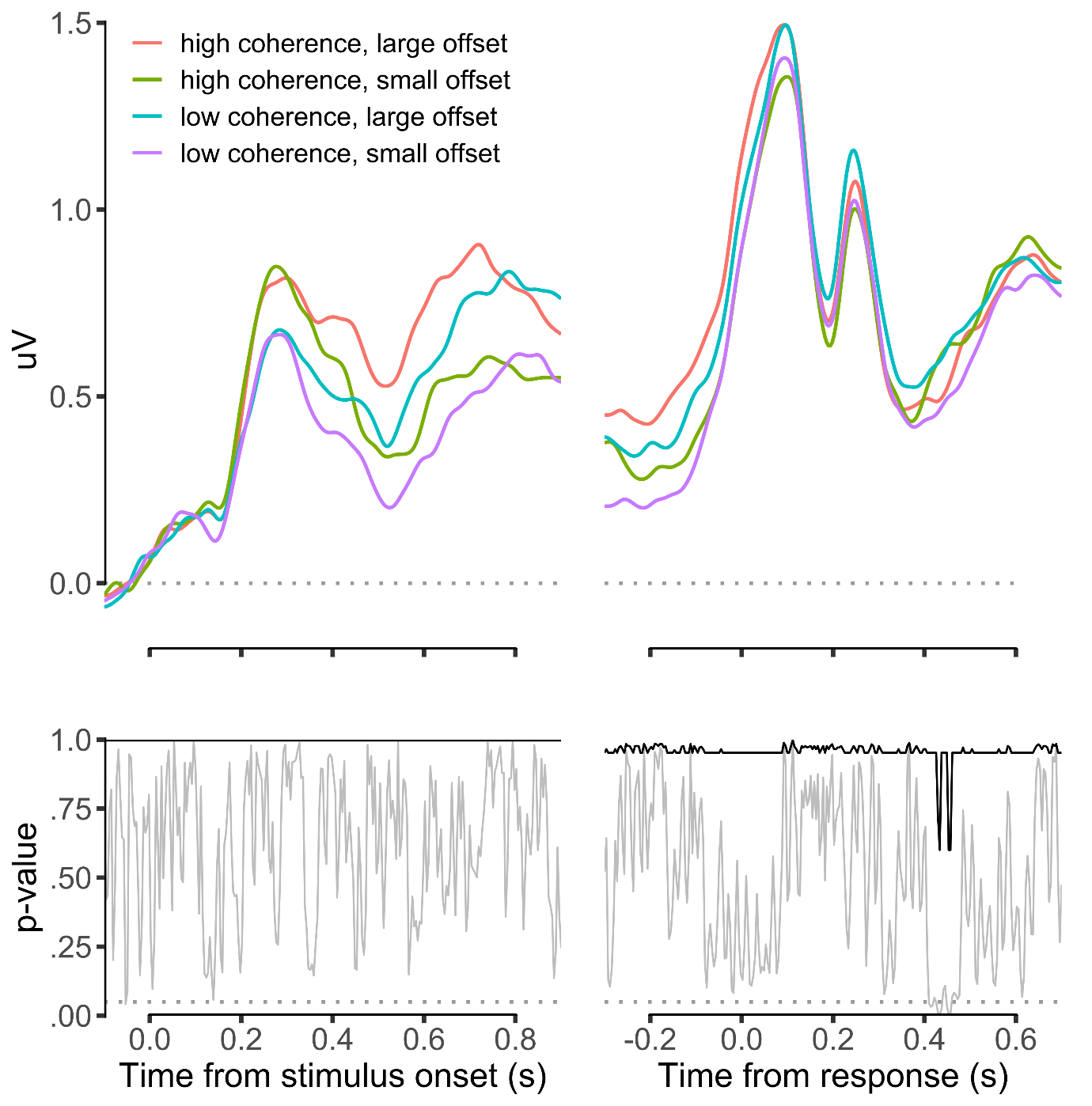


***Figure S2.*** Centro-parietal positivity (CPP; Cz, CPz, Pz, CP1, and CP2 electrodes) spilt by Coherence and Offset for stimulus-locked (left) and response-locked (right) epochs. Lower panels show raw (grey) and false-discovery rate corrected (black) p-values for the interaction effect.
